# Shared Sanitation Management and the Role of Social Capital: Findings from an Urban Sanitation Intervention in Maputo, Mozambique

**DOI:** 10.3390/ijerph15102222

**Published:** 2018-10-11

**Authors:** Tess Shiras, Oliver Cumming, Joe Brown, Becelar Muneme, Rassul Nala, Robert Dreibelbis

**Affiliations:** 1 Department of International Health, Johns Hopkins Bloomberg School of Public Health, Baltimore, MD 21205, USA; tess.shiras@gmail.com; 2 Department of Disease Control, London School of Hygiene and Tropical Medicine, London WC1E 7HT, UK; Oliver.Cumming@lshtm.ac.uk; 3 School of Civil and Environmental Engineering, Georgia Institute of Technology, Atlanta, GA 30332, USA; joe.brown@ce.gatech.edu; 4 WeConsult, R. Fernando Ganhão, Maputo 1103, Mozambique; bacelar@we-consult.info; 5 Instituto Nacional de Saúde, Av. Eduardo Mondlane 1008, Maputo 1101, Mozambique; rassulmn@gmail.com

**Keywords:** sanitation, shared sanitation, collective action, social capital, urban

## Abstract

Shared sanitation—sanitation facilities shared by multiple households—is increasingly common in rapidly growing urban areas in low-income countries. However, shared sanitation facilities are often poorly maintained, dissuading regular use and potentially increasing disease risk. In a series of focus group discussions and in-depth interviews, we explored the determinants of shared sanitation management within the context of a larger-scale health impact evaluation of an improved, shared sanitation facility in Maputo, Mozambique. We identified a range of formal management practices users developed to maintain shared sanitation facilities, and found that management strategies were associated with perceived latrine quality. However—even within an intervention context—many users reported that there was no formal system for management of sanitation facilities at the compound level. Social capital played a critical role in the success of both formal and informal management strategies, and low social capital was associated with collective action failure. Shared sanitation facilities should consider ways to support social capital within target communities and identify simple, replicable behavior change models that are not dependent on complex social processes.

## 1. Introduction

Globally, 2.3 billion people lack access to improved sanitation facilities [1]. Of these, 600 million people use ‘improved’ but shared latrines [1]. According to the World Health Organization and United Nations Children’s Joint Monitoring Programme (JMP), all improved sanitation facilities shared between two or more households are defined as ‘limited’ sanitation services [1]. For the purposes of this paper, we will refer to ‘limited’ sanitation services as ‘shared improved’ sanitation. Shared improved latrines are most prevalent in urban sub-Saharan Africa, used by 18% of all households [1].

Nested within a health impact evaluation of a shared sanitation intervention in Maputo, Mozambique, this research qualitatively examined access, use, and maintenance patterns among intervention (shared, improved latrine users) and control (shared, unimproved latrine users) users. We compare the formal and informal characteristics of existing latrine management systems and examine the formal and informal processes that define current management systems and the impact these systems have on facility quality and use. 

Maputo has a population of approximately 1.2 million people, 70% of whom live in informal slum settlements [2], although population-based surveys often under-estimate the proportion of urban poor who live in slum communities [3,4]. Currently, Maputo has a mean population density of more than 4000 people per square kilometer, varying significantly across neighbourhoods. Between now and 2050, approximately two-thirds of the country’s future population growth is expected to occur in cities; a trend that will put additional stress on already inadequate public health infrastructure and further contribute to the “urbanization of poverty” [5]. An estimated 89% of all Maputo residents use on-site fecal management systems (non-sewered), and only 26% of all fecal waste in the city is safely disposed of, treated or safely managed [6]. 

Population growth and sanitation coverage levels are similar in many of the rapidly growing cities throughout Sub-Saharan Africa, where population growth has far outpaced the expansion of urban services [1]. Efforts to improve sanitation access and use in densely populated urban areas must account for lack of centralized sewerage networks, limited space, and high rates of urban poverty. Shared sanitation services may be an important intermediary step towards meeting Sustainable Development Goal targets for universal sanitation coverage. However, studies show that shared latrines are often poorly maintained, resulting in unhygienic conditions that dissuade regular use [7,8,9,10]. The low rates of maintenance and upkeep can be viewed as a Tragedy of the Commons: a scenario in which the benefits of a shared good are maximized by each individual, where the common good becomes depleted over-time because associated costs (e.g., a dirty latrine, bad smell, blocked septic tank) are borne by all users [11]. 

Literature suggests that collective action is necessary to overcome the tragedy of the commons [12]. For shared sanitation, this collective action includes formal or informal management systems that regulate sharing, cleaning, and maintaining common latrines. When collective action is absent or insufficient to maintain a shared good, it is referred to as a collective action failure. Social relationships and social networks are important factors in shaping how individuals manage or behave with respect to a common good [13], yet there is limited research regarding the influence of social relationships on shared sanitation collective action or collective action failure [14,15]. Various constructs have been defined that describe the types of interpersonal relationships among a group of people and how those relationships determine or shape collective processes. Our research focuses specifically on the concept of social capital, defined as the amount of trust, support, or cohesion that exists among a group of individuals within a specific community [16]. Our study sought to explore the differences in management processes between users of improved and unimproved shared latrines and investigate the determinants and impacts of collective action processes. Conducting this study in an intervention setting allowed us to compare between improved and unimproved shared facility users as well as beneficiaries who had transitioned from using an unimproved to an improved shared latrine in the past year. 

We defined management as the systems, processes, and rules—either formal or informal—that dictate who can and cannot use latrines, who is and is not responsible for maintaining facilities, and who enforces rules at the compound level. Maintaining facilities included multiple processes, including latrine cleaning, repairing or replacing infrastructure, and fecal sludge management (FSM). FSM refers to the removal of fecal waste from latrine sites and can be done manually or machine-facilitated and can be done by the users or through a variety of formal and informal service providers. We focused our inquiry on FSM at the compound—exploration of fecal management chains after leaving the compound was beyond the scope of the present study.

## 2. Materials and Methods

### 2.1. Study Site—Intervention Description and Setting

This study was nested within the larger MapSan health impact trial, a controlled before-and-after (CBA) study to evaluate the effect of an improved shared sanitation intervention on child health (clinicaltrials.gov NCT02362932 [17]). Water and Sanitation for the Urban Poor (WSUP), an international development non-governmental organisation (NGO), implemented the sanitation intervention between March 2015 and March 2016. WSUP implemented the intervention in 11 *bairros* (neighborhoods) in peri-urban informal slum settlements in Maputo City. In these *bairros*, groups of small houses—made from wood, corrugated iron sheets, or concrete—are clustered together around a small, multi-purpose shared space used for cooking, cleaning, playing, and working. Each set of houses and its shared space is called a compound and is often delineated by a wall or fence. Traditional, unimproved shared latrines may be found inside or just outside of a compound’s defined space. Compounds are connected to one another by several car-accessible dirt roads but primarily by narrow, winding pathways. Each compound has a chief, *chefe*, who is an informal leader of the compound residents and may help to structure latrine use and management. 

The intervention included construction of pour-flush toilets (some squat and some with pedestals) with a septic tank that are shared among compound residents. These latrines have two variants: (1) a Shared Latrine (SL) is a single latrine unit, designed for compounds with 20 or fewer residents; and (2) a Compound Sanitation Block (CSB), designed for compounds with more than 20 residents, with a cubicle for every 20 people (images included in Supplementary Materials). Both SLs and CSBs are constructed with cement blocks and have a metal door; all are handicapped accessible. CSBs are constructed with a piped water connection to a water storage tank and tap on the outside of the latrine; users, however, must organize and pay for access to the municipal water authority. CSBs also include a rain harvesting storage tank connected to a water tap inside the latrine and a clothes-washing station. Both SLs and CSBs have a drain and a sink basin on the outside of the structure. Intervention latrines (improved, shared) are situated within each compound’s shared space, with the specific location determined by engineering considerations and community preference. Latrines often occupied the space of the old pit latrine. Thus, the latrine position within the compound varies: it could stand near the entrance, extremely close to one, or several, homes, in a corner, or more centrally located in the shared space and further from individual houses. At the time of construction, WSUP worked with compound residents to develop management and operations systems for the compound. 

Control compounds have traditional (pit) shared latrines (TLs). TL compounds included a range of facility quality, from rudimentary facilities with cloths hanging as walls and no roof to otherwise improved facilities that include concrete slabs. The original position of the latrine within these compounds is normally in a corner as far as possible from the households. However, due to poor fecal sludge management, when a latrine becomes full, a new one is constructed. Given limited space in the compound, TLs are moved and, over time, are constructed within the compound’s shared space extremely close to households.

### 2.2. Sample & Participant Selection

We selected participants from four types of compounds. From compounds participating in the MapSan Trial, we selected: (1) compounds with traditional shared latrines (TL); (2) intervention compounds that received and share an SL as part of the MapSan study; (3) compounds that received and share a CSB as part of the MapSan study; and (4) compounds that received a WSUP-constructed SL or CSB three or more years prior to the MapSan intervention (between 2009 to 2014, referred to as Older Intervention Latrines [OILs]). OIL were selected to learn more about changes in latrine maintenance and management processes as well as user satisfaction over time. Please see Brown et al. [17] on efforts taken to ensure comparability between control and intervention groups. The study sample aimed to include 75% women and 25% men, as women are responsible for the majority of sanitation-related tasks and are likely to spend more time in the compound [18]. 

### 2.3. Data Collection

A five-person data team was trained by the principal investigator and research manager in qualitative methods and conducted 24 unstructured observations, 96 in-depth interviews (IDIs), and 7 focus group discussions (FGDs). Interviews were conducted in either Portuguese or Changana, depending on the respondent’s native language. Methods were informed by Grounded Theory [17] and data collection and analysis were concurrent. Research questions and research guides were adapted on an iterative basis as new themes emerged and as data collectors reported on lessons learned in the field. We terminated sampling once the research team determined that the data had reached theoretical saturation, the point at which themes are repeated and no new information appears in the data [19]. Data collection methods used in the present study included:

IDIs: Interview participants were recruited at the compound level. The data collection team selected interview participants purposively to fulfill the gender and compound type quotas. Each interviewer followed prompts from an open-ended guide to learn about the respondent’s sanitation experience, compound management processes, and changes in latrine use and maintenance over time—particularly in the case of intervention beneficiaries. Each interview took 30–60 min. 

FGDs: Each FGD included a group of residents of the same gender and age group who used the same type of latrine. FGDs focused on comparing residents’ sanitation experiences and differential latrine management processes. Some FGDs included only compound chiefs, who were well-positioned to discuss strategies to facilitate latrine management. FGDs had between 5–12 people, summing a total of 47 participants. Some individuals who participated in a focus group also participated in an IDI and/or observation.

Initial IDI and FGD guides have been included as Supplementary Materials.

### 2.4. Data Analysis

All interviews and focus groups were audio recorded, transcribed verbatim, and translated into English by the data collection team. The data team provided detailed summaries of unstructured observations including a chronology of observed activities. All data was analyzed concurrent to data collection. The study team did not use an existing theoretical framework or set of assumptions to guide analysis. Rather, the team analyzed data inductively by reading transcripts, summarizing data collection events, holding frequent team debriefs, and conducting initial line-by-line coding to identify relevant themes. These processes allowed the research coordinator to write thematic memos and develop an analytic codebook, which was used to code all data with *NVivo* software (Version 10, 2014; QSR International Pty Ltd., Melbourne, Australia). Collecting and analyzing data simultaneously allowed the research coordinator to identify new themes as they emerged from the data and include additional probes in order to elicit further context around these themes. 

### 2.5. Ethical Approval

All interview and focus group participants provided written informed consent prior to data collection. All interview and focus group participants were at least 18 years of age. Data collectors informed participants of their right to end the data collection event at any time or skip any questions. Compound chiefs were provided with and signed a participant information sheet prior to observations but did not sign a written consent form, as observations occurred in public spaces. No personally identifiable information was collected on data collection tools and names or other identifiers included in audio recordings were redacted during transcription. All audio recordings were permanently deleted. 

This study received approval from the Ethics Committee of the London School of Hygiene and Tropical Medicine’s (Reference 11791) and from the Comité Nacional de Bioética para a Saúde (IRB00002657) do Ministério da Saúde, República de Moçambique.

## 3. Results

The data team collected 96 interviews, 70 with women (73%) and 26 with men (27%); 27 IDIs were with CSB users, 26 with SL users, 27 with TL users, and 16 with users of older intervention latrines (OILs) (Table 1). Of the seven FGDs, three were with *chefes* from SL, CSB, and TL compounds. 

### 3.1. Latrine Management Strategies and Collective Action Failure

Just over one-half of IDI participants from intervention compounds reported that their compound had an organized cleaning process for managing latrines. In comparison, only one-third of traditional latrine users noted that their compound had a cleaning system. Processes ranged from a very formal rotation among families with specific female family members tasked with cleaning duties to an informal system without a defined schedule. In compounds with an informal system—which were more likely to exist in TL compounds—residents reported that whomever woke up first would typically clean or that cleaning responsibilities fell to the compound *chefe*. Among compounds that had a cleaning system in place, approximately 70% of IDI participants reported that compound members adhered to the schedule. 

Collective action failures described in interviews typically focused on neighbors who did not do their part in keeping the toilet clean, including cleaning up after their children. A small number of intervention participants (less than five) complained that community members would not bring water to wash down urination, which created bad smells in the latrine. These respondents were notably from households located close to the latrine. Some participants (*n* = 6), mostly from intervention compounds, reported collective action failure due to lack of financial resources. When it came time to collect money for ongoing maintenance or for water payments, same families said they did not have enough money to contribute. This lack of contribution was a source of strife in the compounds.

We noted variation in latrine management system based on the type of facility available in compounds. Compound Sanitation Blocks (CSBs), which have a larger number of users, were on the extremes of sample variability. Roughly two-thirds of CSB users reported that their compound had a management strategy in place. Management strategies in place were typically more complex than other latrine users and included dividing the latrine units into male or female, allocating particular latrine units to a set group of families, holding monthly compound-wide cleanings, organizing a monthly financial contribution for ongoing maintenance, or using keys to regulate use. Only a small number of CSB users (*n* = 4) reported few or no rules guiding latrine use and maintenance. While rare, these users reported that the lack of rules resulted in extreme unhygienic conditions or conflict given the larger number of residents sharing the latrine. 

Traditional latrine users were least likely to report having a management strategy in place. Most TL participants who did report a cleaning schedule said that this was based on a mutual understanding of how to respect the bathroom rather than based on an agreed-upon schedule. Lack of formal cleaning systems did not necessarily translate to lack of maintenance and repair of facilities. A few TL users (*n* = 3) who reported that there was no formal a cleaning system said that compound members could and did come together to contribute financially to maintenance or when something in the latrine needed repair. Notably, this process of engaging residents to contribute for sanitation repairs was typically led by the compound *chefe*. 

There were no substantial differences in latrine management strategies between newer and older shared improved latrine compounds. In some OIL compounds, management strategies had weakened over time. However, others noted that their compounds had grown more organized over time with more detailed cleaning schedules, monetary collection, and specified water use protocols such as charging a small fee to collect water from the shared tap.

Approximately 40% or CSB users reported independently that they are happy with their latrine management structure and the way that the compounds residents use the bathroom. Very few SL and TL participants (*n* = 3) reported independently (i.e., without probing) during interviews that they are pleased with the current management structure and that there are no problems among their neighbors. 

There was a direct link between the ways respondents described management strategies and the perceived quality of their own latrines. Respondents that described weak or informal management systems also viewed their latrines as dirty, disgusting, or unhygienic.

*It is annoying when you want to use the bathroom and it is dirty. In a bathroom in which many [people] use, it should be very hygienic, but this doesn’t happen here. Sometimes I am hesitant even to enter because I think that I could see poop, but I have to gain strength and use because I don’t have a choice.*

—CSB User

*Unfortunately, in my compound, there’s no organization on the part of residents according to the care of cleaning, some do cleaning but others do not, and this creates certain nausea when entering into the latrine.*

—SL Focus Group Participant

Newly constructed latrines were viewed by some as more difficult to maintain than TLs. Dirt and unhygienic conditions were more apparent in the high quality new facilities, requiring more frequent cleaning and maintenance. In addition, the new latrines require the use of water to wash down urine and fecal matter. This is a new process for intervention users: water is not used in most TLs, as it fills the pits more quickly. In some compounds, the high visibility of dirt and use motivated individuals to follow new cleaning processes to keep the latrine in good condition. However, in others, this challenge was a source of tension among neighbors.

*Now what worries me is the union between us. I always say that after urinating we have to put water, but people don’t do it…I say if someone doesn’t have the liquid [soap] to wash the bathroom, they can use [detergent], because it is difficult to wash the bathroom only with water…even now when it is “cleaned”, I just open the door and I feel the smell I don’t know what is to be done.*

—CSB *Chefe* Focus Group Participant

Across both intervention and traditional latrines, management and maintenance of latrines followed gendered lines. Women were typically responsible for cleaning all facilities and men were responsible for repairs and fecal sludge management. In SL and CSB compounds, newer latrines meant that significant repairs and fecal sludge management had not emerged as a concern yet. For TL users, however, fecal sludge management was a source of tension. Most respondents relied on manual fecal sludge removal followed by digging a new pit. Respondents viewed FSM as difficult, dirty, and costly. In smaller compounds, it was problematic to find space for a new pit, so individuals opened up old pits after some of the fecal sludge had sunk into the soil. Moreover, respondents complained of the horrible smell that proliferated during desludging. Most OIL users (*n* = 12) also had not emptied their septic tanks yet. The few that had done so contracted a private community company and collected money from each household to pay for it. 

### 3.2. Social Capital: A determinant of Successful Latrine Management 

Research participants often expressed proxies for social capital by saying that there was “understanding” among compound residents or that individuals treated one another “like sisters and brothers.” Conversely, when latrines were mismanaged, individuals reported low social capital by casting blame on neighbors or telling stories of arguments among women in the compound due to lack of cleaning.

*In my compound people do not accept cleaning the house [latrine]. They deny to do it…. in my house, we are not even talking to other people because of the cleaning problem.*

—SL User

Participants reported financial barriers to optimal shared sanitation management. Latrines require funds for ongoing maintenance, including repairs and fecal sludge management. This presents a challenge with shared sanitation facilities, particularly among low-income households where saving is difficult. Interview respondents in all four compound types reported the difficulty in collecting funds for latrine maintenance or water bills. Some compounds organized a monthly financial contribution for ongoing latrine maintenance. However, such a fund was not successful without a sufficient degree of social capital. In one case, compound members reported that they had contributed to a latrine fund for over a year and when the money was needed for desludging, the compound leader reported that the money was gone. The respondent noted a lack of trust and understanding regarding the use of this money. Lack of transparency and miscommunication about sanitation funds can impact the cycle of decreased social capital and collective action failure. 

High social capital was also reflected through community norms and adherence to an agreed-upon cleaning and maintenance schedule. Some participants discussed a compound-wide understanding about how and when to clean the latrine, demonstrating a mutual commitment to latrine quality as well as a mutual respect for one another by taking care of their shared good.

*P: We are organized in groups of three people per day to take care of the whole sanitary block.*

*I: And if someone refuses?*

*P: Nobody can refuse, we have a law here… Never happened that someone refuse cleaning up here, that is way this bathroom never smelled bad, people all know when and who should do the cleaning.*

—CSB User

### 3.3. Locus of Control

External locus of control is a concept to describe when individuals feel that they lack personal agency to make changes. Participants frequently displayed an external locus of control attitude toward latrine maintenance. Participants expressed how difficult it was to encourage others to pitch in and help clean the latrine or contribute financially for ongoing maintenance. Further, many participants (*n* = 55) reported that they felt disempowered to proactively discuss latrine maintenance with neighbors, explaining that they did not feel that it was worth it to make “noise” in the compound by bringing up potentially contentious issues. Additionally, several younger participants (primarily females) justified their hesitation based on social hierarchy. For example, one SL IDI respondent noted that she had ideas about ways to improve menstrual hygiene management and use of the latrine, but she noted that she didn’t make suggestions:

*I am new here; there are things that I think and keep to myself, it is not my place.*

—SL User

The external locus of control impacted collective action. Some respondents (*n* = 12) were hesitant to ask neighbors to clean the latrine in accordance with agreed systems. Speaking out could have negative repercussion on community relations and social capital.

*We sometimes wait 2 or 3 days hoping they’ll [neighbors] wake up and see that [the latrine] is dirty, but we see that nobody washes, so we take brooms and wash…I can’t say anything because I’m not a boss. The mama who is boss here can’t stand the dirtiness, but when she talks, they say that she talks a lot, that she’s crazy.*

—*CSB User*

Participants who felt that there was little trust or social capital among their neighbors also felt that they did not have the ability to ask their neighbors for support or to change their latrine cleaning behavior. In this way, a vicious cycle can be created where low social capital leads to collective action failure which perpetuates conflict among neighbors and furthers lack of social capital. For example, one CSB compound *chefe* who did feel empowered to instruct neighbors on sanitation cleaning and maintenance reported that this “noise” had created more conflict among compound members:

*…[my] noise is why we do not understand each other because I talk too much and then they hate me.*

—CSB *Compound Leader*

In some compounds, individuals that embodied an *internal* locus of control attitude attempted to hold meetings and talk with residents to drive effective group decision-making and collective action. At times this was effective, such as one woman who rallied a large portion of women from a 150-person compound to participate in monthly CSB cleaning in addition to their regular cleaning. However, this woman was a ‘positive deviant’: her behavior was uncommon compared to her peers who faced the same collective action issues. Yet, she employed particular strategies to unite neighbors and successfully maintain the shared CSB. In contrast, most interview participants mentioned that when compound residents tried holding meetings to discuss cleaning or maintenance techniques that behavior change was not sustained, and people stopped adhering to agreed-upon strategies. Difficulty in collecting financial contributions from each household for latrine maintenance also contributed to an external locus of control attitude. Intervention and control users discussed ideas for better managing latrines, but noted that this was simply not feasible given lack of funds either from their own household or from neighbors. 

### 3.4. Factors That Affect Collective Action Failuire

Collective action failure was more frequent in compounds where more houses shared the latrine. Approximately 25% of people from compounds with five or fewer households sharing the latrine reported significant management issues and feelings of stress compared to half of individuals from compounds with six or more households sharing the latrine. Accordingly, latrine management issues were also more frequent in CSB compounds (which require at least 20 users by definition). Approximately 2/3 of CSB users reported feelings of stress related to management of the shared facilities. Over time, users of older CSBs reported that attention to latrine cleanliness and maintenance had diminished.

*When we locked [the latrine] it was well maintained because the person came and asked for keys, there was someone responsible for the keys…but now everything is bad… Our bathroom, it’s not well preserved because anyone enters in anyway, there are people who enter and don’t put water, they dirty the bathroom [defecate] and don’t put water and when you get there, you find that dirt and it is bad.*

—User of Older CSB

*P: There are two bathrooms, and we are 10 houses and each bathroom has 5 families, and of the 5 houses only one person washes and here out of 4 houses only one person [washes], we can’t stand it.*

*I: And when you talk to these people?*

*P: They don’t respond, they don’t do anything, it is only us who doesn’t like dirt, so we wash.*

—User of Older CSB

The low social capital/collective action failure relationship was most common in CSB and TL compounds, while SL compounds were less likely to experience low social capital. SLs are used by fewer residents, often making it easier both to maintain the unit as well as to relate to and accommodate neighbors. In fact, some SL compounds may have several households that are all familial relatives. Individuals in larger TL or CSB compounds discussed how difficult it was to share sanitation facilities with so many other people, explicitly connecting the size of the compound to the difficulty in organizing individuals and collectively understanding one another and functioning as a cohesive group. Poor construction materials, broken slabs, and unsealed pits often meant that TLs were dirtier and less hygienic than newer latrines, reducing individuals’ incentives to maintain facilities or participate in cleaning schedules. 

The relationship among low social capital, collective action failure, and compound size was potentially mediated by specific management and access to shared facilities. For example, in one large CSB compound with 70 residents, the latrine units were divided among specific groups of families and there was an organized and adhered to cleaning schedule in place.

*P: …we take care [of the latrine] so that it can last many more years…every house does the cleaning job and this helps us a lot to keep the bathroom clean. In this compound lives more than 70 people sharing the same bathroom, if we do not keep it well cleaned, it will get very horrible and smelling bad.*

*I: Who determined that each family will do the cleaning job per day?*

*P: We ourselves; we all get involved and we all feel like the owners of the latrine.*

However, later in the interview, the participant remarked on the lack of friendliness among neighbors:

*Here in the compound we are neighbors but we are not very friendly, there are many discussions for so small reasons, we live distrusting of each other, much intrigue, envy, gossip. Sometimes children play then get hurt, and parents argue loud and ugly, sometimes someone throws dirty water in front of your house on purpose just to argue.*

—CSB User

While this low social capital didn’t affect latrine cleaning, the residents did have trouble organizing financial contributions for water. It is clear, though, that factors other than social capital are important motivators and determinants of latrine collective action. Such motivators could include perceived value of latrine cleaning, as evidenced in the quote above, or increased social status from a clean latrine, as evidenced from the quote below:

*…the bathroom doesn’t belong only to the people who live in this house but to anyone…even those who pass by on the street can ask for the bathroom and if it is dirty is a shame for ourselves*

—*CSB User*

Some compounds used shame and social sanctions as an accountability mechanism to motivate latrine maintenance. For example, one SL user noted that if a family neglected their cleaning responsibility, the latrine leader locked the bathroom and prohibited them from using the latrine. She went on to say that “the family redeemed itself and nobody else ever refused to clean it, that’s our protocol.” Another participant noted that their compound leader died and no one succeeded him, leading to latrine mismanagement and an ad-hoc cleaning schedule. Thus, having a leader in place to drive collective decision-making and accountability may be an important determinant of latrine maintenance.

Data suggest that other factors were important determinants of latrine management that are likely associated with some aspect of social capital. For example, three participants said that renters, who were often more transient compound residents, did not do their part in latrine maintenance.

## 4. Discussion

Approximately half of all respondents reported that their latrines had formal management or cleaning structures in place. Management structures ranged from formal to informal systems for sharing and maintaining the quality of sanitation facilities. Latrine management was directly linked with perceived quality of latrines.

Although intervention delivery included engaging with local compounds on management and helping to establish systems for the maintenance and upkeep of facilities, only half of residents reported formal structures. In dynamic urban environments, building and maintaining collective processes may be challenging, even in small compounds. Our data suggest that current strategies have low-reach among the intended population, are still subject to collective action failures, and become a source of stress among residents [20]. However, there are few published studies exploring the impact of interventions specifically targeting shared sanitation and use. Tumwebaze and Mosler [21] found a positive effect on toilet cleanliness associated with a discussion and commitment-based intervention in Uganda; however, this intervention model still requires long-term engagement with users and may face the same barriers that current strategies demonstrate in reaching all users. Alternative, less labor-intensive models for developing and maintaining cleaning and maintenance systems are needed.

Within the context of managing and maintaining a shared latrine, the constructs of social capital, collective action failure, and an external locus of control are closely connected and, together, help explain common experiences for managing or mismanaging shared sanitation facilities. Latrine mismanagement was a very common source of stress and neighborhood conflict among research participants in Maputo [20]. The ability to organize and respect a latrine maintenance process was largely dictated by the degree of social capital within the compound. Latrine collective action may have been mediated by other determinants, such as a desire to use a clean latrine, the social status associated with having a clean latrine, or having a compound leader. Despite those factors, though, compounds with functional management systems in place exhibited some degree of trust and support among neighbors to collectively maintain their shared commodity and avoid a tragedy of the commons. 

Our finding regarding the key impact of social capital on shared sanitation management is supported by a 2014 study on collective latrine cleaning in Ugandan slums that measured proxies for social capital and collective action [10]. The study asserted that users who positively associated with other users, talked easily with other users, and felt they were part of a team with other users were significantly more likely to have higher commitment to cleaning [10]. Additionally, the study found that cleaning commitment was higher among residents who believed that cleaning was dependent on collective cooperation among users [10].

When management systems were not in place or adhered to, social capital, locus of control, and collective action failure often negatively impacted one another creating a vicious cycle. Our results show that lack of ‘understanding’ led to collective action failure and an unhygienic latrine, creating stress and neighborhood conflict. Given the low degree of social capital, efforts to talk and problem-solve were often reported to be fruitless. Simiyu et al.’s recent research in Kisumu, Kenya on determinants of shared sanitation quality found that meetings and collective decision-making were more effective in compounds with fewer households or in those with a leader present [9]. Our findings support this conclusion, leading to a larger discussion on the role of compound size and the potential of collective action.

While the number of households sharing the latrine does correspond to the total number of people sharing the latrine, they are not completely synonymous. For example, a compound with six households may have anywhere from 12 to 35 people living in it. The number of people sharing a latrine may complicate latrine coordination; however, individuals within each household unit are typically family members and tend to have high social capital. Thus, collective action issues in larger compounds appear to stem from a high number of distinct households that lack ‘understanding’ or social capital, rather than the number of people. Quantitative approaches to test the relationship between social capital and latrine management will help better understand the ways in which social processes determine the quality and nature of shared resources. There are a range of tools available for measuring social capital and related constructs, although only a limited number of these have been tested and validated in low-income settings [9,10]. Quantitative research to measure social capital would help to better quantify the relationship between social capital and sanitation quality and test a range of hypotheses generated by this work. One specific example is exploring how social capital mediates the relationship between shared sanitation quality and the number, type, and relational structure of users.

We recognize a specific limitation to our research. This research used purposive sampling and captures the experiences of women and men using shared sanitation facilities in urban Mozambique. Findings may not be generalizable outside the specific geographic and cultural context. However, key discussion themes may resonate among shared sanitation users in other urban, East African settings. Many of our respondents had received new latrines less than a year before data collection. Improved facilities were generally high quality and larger scale maintenance needs had not emerged. In addition, few improved latrines had reached storage capacity, reducing participant direct experience with FSM or emptying septic systems. Data collection did not include systematic assessments of latrine cleanliness, as we did not want study participants to feel as though they or their latrines were being evaluated. Thus, we are unable to triangulate if existence of a latrine management or maintenance structure resulted in observed latrine quality or cleanliness. Social capital as a determinant of successful latrine management was not an *a priori* research hypothesis, but a finding that emerged during analysis and review. While there is some indication that specific social relationships impact views on latrine management—such as the view that renters did not contribute to latrine maintenance—and we were unable to explore some of these factors within the specific context of social capital. How social capital is shaped by length of tenure, familial relationships, and similarities in resident background should be explored or accounted for in future research. Our sampling strategy was purposive, and findings may not be generalizable beyond participants with similar characteristics. Further, the nature of qualitative inquiry relies on individuals who are willing to participate in the research process and reflect on their own experiences. We included adequate training on rapport building and probing for our data collection team in order to ensure that our data reflected the broad range of experiences among our sample.

Despite these limitations, this research has important implications for future sanitation programming. While shared facilities will not count towards Sustainable Development Goal targets, they are an unavoidable reality for high density urban populations in the immediate future [1]. Ensuring that facilities are adequately maintained and that interventions are responsive to the complex social processes that influence maintenance and upkeep—coupled with strategies for safe conveyance and disposal—are necessary to ensure that these shared facilities adequately prevent exposure to fecal waste and provide the necessary dignity, privacy, and accessibility to users. 

## 5. Conclusions

From a programmatic perspective, developing social capital within small community units is a challenging task to increase shared sanitation collective action strategies. However, numerous compounds of various sizes have successfully managed their shared latrines. Drawing from a positive deviance approach, WASH interventions could learn from compounds that are employing effective collective action strategies to disseminate lessons learn and share behavior change tactics. Based on our research, such tactics might include electing a compound leader to implement and oversee adherence to latrine management strategies, holding monthly compound meetings to increase latrine ownership and collective decision-making, and creating a monthly financial contribution to help with ongoing latrine maintenance costs or cleaning supplies. Simple, low cost interventions informed by modern behavioral science may also provide replicable approaches for increasing social capital or finding mechanisms for latrine management that rely less on complex social processes. 

## Figures and Tables

**Table 1:** Data collection summary.

Latrine Type	In-Depth Interviews (*n* = 96)	Focus Group Discussions (*n* = 7)
Traditional Latrine (TL) User	27 (28%)	1 (14%)
Shared Latrine (SL) Users	26 (27%)	4 (57%)
Community Sanitation Bloc (CSB) Users	27 (28%)	2 (28%)
Older Improved Latrine (OIL) Users	16 (17%)	0 (0%)
Total	96 (100%)	7 (100%)

Note: Percentages may not add up to exactely 100%, owing to the rounding off.

## Supplementary Materials

The following are available online at http://www.mdpi.com/1660-4601/15/10/2222/s1. A: In-Depth Interview Guides, B: Focus Group Discussion Guides.
