# Supplementary material for: Shared Sanitation Management and the Role of Social Capital: Findings from an Urban Sanitation Intervention in Maputo, Mozambique
Source: Int J Environ Res Public Health. 2018 Oct 11;15(10):2222. doi: 10.3390/ijerph15102222 (PMC6210686; doi:10.3390/ijerph15102222)
Supplement: Supplementary file 1 [file ijerph-15-02222-s001.pdf]

Supplemental Information:

A. In-Depth Interview Guides

B. Focus Group Discussion Guides

A – IDI GUIDE

After obtaining oral or written consent and permission to record the interview, proceed with this introduction and the questions below.

Thank you for participating in the interview today. My name is ____________. I work with WE Consult and the Ministry of Health. Thank you for meeting with me today.

The following questions serve as a guide for discussion but can be flexible if the conversation topics remain broadly on shared sanitation and community well-being related to sanitation.

Introductory Questions:

1. Could you tell me about your experience with the sanitation facility near your house?
2. What do you like or dislike about the sanitation facility?
    - Probe: What would improve your sanitation facility?
3. What would be your preferred/ideal sanitation option? Why?
4. Have you or others in your compound received hygiene promotion or sanitation education? Could you describe this?

For intervention compounds:

5. What did you use before the sanitation facility was built?
6. How does the old toilet compare to the sanitation facility that is here now?
7. Is the old toilet still here? If so, do people still use it? What are the reasons for this?

Attitudes and Stress

8. How do you feel about using the sanitation facility?
9. Does the sanitation facility in your community ever make you feel unsafe?
    - Probe: How do you use the latrine at night? How do others in your compound use the latrine at night?
10. Can you talk about if the latrine offers privacy?
11. Do you have other stress in your life related to the latrine?

For Female Respondents on Menstrual Hygiene Management:

12. How would you explain menstruation to a niece or daughter?
13. Could you explain how you normally manage your period?
    - Probe: Where do you dispose of menstrual management items?
14. Does the sanitation facility accommodate your menstrual management process? Why or why not?
15. How could the latrine be improved to meet your menstrual hygiene management needs?

Patterns of Shared Sanitation Use and Maintenance

16. How are the relationships among compound members?
    - Probe: WHY? (understand why they think people get along or don't get along)
17. How is the sanitation facility managed and maintained?
    - Probe: How was the sanitation facility maintenance process determined?
    - Probe: Can you explain what happens if something breaks in the latrine or is not working properly?
18. Do you have suggestions to improve the maintenance of the sanitation facility?
19. How do you think that people's relationships in the compound affect using and maintaining the latrine?
    - Probe for married individuals: Do you think that being married makes it harder to take care of the latrine?
20. Are there any rules or restrictions about using the sanitation facility? What are they? Who creates these rules?
21. Can you explain what happens when the latrine is full?
    - Probe: How does payment work for this?
22. What is your biggest complaint with your current latrine?

<u>Demographic Characteristics</u>

23. Type of Compound:
    - MapSan Intervention
    - MapSan Control
    - Other compound with traditional latrine
    - Compound with older WSUP latrine
24. Latrine Type
    - WSUP shared latrine-new
    - WSUP sanitation block-new
    - WSUP shared latrine-older
    - WSUP sanitation block-older
    - Flush/pour toilet that drains above ground, open hole
    - Flush/pour toilet with piped sewer system
    - Flush/pour toilet with tank/underground pit
    - Pit latrine with concrete slab
    - Pit latrine without concrete slab
    - Open defecation
25. Gender:
26. Age:
27. Marital status:
28. Number of children:
29. Number of children younger than 3 years:
30. Number of children between 3 and 18 years:
31. Number of people in household:
32. Number of households in the compound:
33. Number of households that share the latrine:

34. Education level:
35. Employment activities:
36. Employment of family members:
37. Own or rent the household:
38. Number of years living in the compound:
39. Is there electricity in your household that regularly functions:
40. Does your family own a bicycle, motorcycle, or car:

B – FGD GUIDE

Ice Breaker: Everyone creates a pseudonym on a note card and introduces themselves using their pretend name and favorite color (or something more creative/appropriate that the enumerators think of)

Questions

1. Let's compare the latrine you have now to the old one. Which latrine is easier to use? Easier to clean? Why?
    a. Do you still use the old one? What are the reasons for using the old latrine?
2. What works well about your strategies for taking care of and cleaning the latrines? How could latrine management be improved?
    a. *If not mentioned:* What happens if something breaks in the latrine?
3. Now let's discuss how you use water in the latrine. Can you describe a typical day of your water-related activities? You wake up…then what?
    a. Tell me about a time when the water stopped running or you did not have enough stored water. Have others had similar experiences? How did that make you feel?
    b. How could your experience getting and using water in the latrine be improved?
4. We understand that when you are menstruating, it is important to clean everything and make sure that men and children do not see blood in the bathroom. Do you agree or disagree with this? Why is this important?
5. When you use the latrine, it's nice to have privacy. What are the differences between how you feel now and how you felt using your old latrine with respect to privacy?
    a. Tell me about a time when you felt embarrassed or shame using the latrine.
6. We know that using the latrine at night can be difficult or feel scary. Tell me, has this gotten better or worse for you since the new latrine was constructed? How has it been for your family and neighbors?
    a. What do you or did you do to help lesson any fears or bad experiences?
7. Now that we've discussed a lot of our current and past experiences with the latrine, what would your ideal latrine look like? If you could design the latrine of your dreams? (Facilitator draws a latrine with everyone's ideal characteristics)
